# Wernicke-Korsakoff Syndrome following Small Bowel Obstruction

**DOI:** 10.1155/2002/702526

**Published:** 2002-11-01

**Authors:** Shoumitro Deb, Richard Law-Min, David Fearnley

**Affiliations:** Department of Psychological Medicine University of Wales College of Medicine Heath Park Cardiff CF14 4XN UK

## Abstract

We report a case of a 64-year-old lady who developed clinical features of Wernicke-Korsakoff syndrome following a laparotomy for small bowel obstruction. Following the operation she developed paralytic ileus and required total parenteral nutrition for one month. A suspected history of average 40 units of weekly alcohol consumption prior to the operation could not be confirmed and the patient did not show any sign of alcohol dependence. Within a few months of treatment with a daily oral dose of thiamine 200 mgs supplemented by multivitamins the patient showed subjective evidence of improvement in confusion, confabulation, and anterograde amnesia, although objective tests showed residual deficits in many areas of cognitive functioning, including immediate and delayed recall of verbal and non-verbal materials, planning and switching of attention.

## 1. Introduction

Thiamine deficiency has been associated with many clinical states including alcoholism, persistent vomiting, lesions of the gastrointestinal tract, prolonged fasting, and malignant disease [20,22]. This may progress to Wernicke's encephalopathy, and often despite replenishment with thiamine, the Korsakoff syndrome [42].

The Korsakoff syndrome is defined as a disproportionate impairment of memory relative to other aspects of cognitive function, resulting from thiamine deficiency [20]. It was first formally described in both alcoholics and non-alcoholics by Korsakoff [22]. The Wernicke's syndrome, named after Wernicke [42], on the other hand is characterised by the acute onset of confusion, ataxia and opthalmoplegia which commonly but not always precede the memory disorder. Subsequently it was found that the two syndromes were in effect different stages of the same disease process and the term Wernicke-Korsakoff syndrome (WKS) was proposed [39].

WKS has since been the subject of numerous studies including case reports and reviews [20]. This syndrome is almost certainly caused by thiamine deficiency [9] and the resulting memory deficit comprises anterograde and severe retrograde amnesia with intact working and procedural memory. Thiamine is converted in the body into an active metabolite called thiamine pyrophosphate (TPP), which is involved in DNA synthesis, glucose metabolism, and the production of neurotransmitters such as acetylcholine, glutamate and gamma-amino butyric acid (GABA). Despite our increased understanding of WKS, it is still unclear why some people are particularly vulnerable to thiamine deficiency, the precise mechanisms by which the brain lesions are produced and the neurochemical abnormalities involved.

Various lesions have been associated with the WKS. Neuropathological abnormalities include petechial haemorrhages, demyelination, gliosis, endothelial proliferation, areas of parenchymal necrosis, general cortical atrophy and neuronal loss [39]. These abnormalities could be found in the paraventricular and peri-aqueductal grey matter, walls of the third ventricle, floor of the fourth ventricle and the cerebellum [8], the mammillary bodies and the thalamus [14]. The critical lesions involved in the impairment of memory have been the subjects of much debate. Some suggested that lesions in the medial dorsal thalamic nuclei

ISSN 0953-4180/01/02/$8.00 © 2001/2002 – IOS Press. All rights reserved cause the psychopathology [21]. However, Victor and colleagues [39], found that all their Wernicke's subjects, with or without subsequent memory problems, had lesions of the mammillary bodies. Lesion of the thalamus was implicated only for those with persistent amnesia. However, only certain thalamic lesions appear to lead to amnesia, such as the anterior thalamus involving the mammillo-thalamic tract [21,24,27,40]. The evidence regarding memory deficit suggests that it is caused by damage to a circuit comprising the hippocampus, entorhinal and perirhinal cortex, the mammillary bodies, mammillo-thalamic tract and the anterior thalamic nucleus. It is argued that the memory loss in the WKS patients is caused by the 'disconnection' of a critical circuit running between the temporal lobes and the frontal cortex [21].

Although frequently seen among alcoholic patients [39], WKS may also occur in non-alcoholic patients with impaired nutrition. In the literature, cases have been described following gastrointestinal lesions such as peptic ulcer, acute pancreatitis, appendicitis, oesophageal metastases, malabsorption syndromes and gastrointestinal surgery [32]. Other causes include prolonged intravenous therapy in postoperative period [12, 29], malignancy, haemodialysis, hyperemesis gravidarum [31], self-induced starvation [1], anorexia and vomiting [3], and transplant surgery.

Surgical removal of portions of the gastrointestinal tract is a known predisposing factor for the development of WKS. It may occur following gastrectomy for neoplasm [2], subtotal colectomy for ulcerative colitis [38], and gastric reduction or diversion operations for morbid obesity [10,30,33,34]. When it occurs, it is usually within weeks (between 4 and 16 weeks), for example, after gastric plication [13,23], although cases following a longer interval (2–20 years) after surgery have recently been reported [37]. The signs and symptoms of WKS are usually a combination of peripheral and central neuropathy and include mental impairment, lateral nystagmus, loss of deep tendon reflexes, weakness and paraesthesia of the extremities and ataxia. Although WKS usually occurs acutely, it can also have a slow onset [19].

It has been reported that the WKS may follow prolonged infusion therapy in the post-operative period and this may have implications for the prophylactic measures particularly after bowel surgery [39]. To highlight this association, we present here a case of WKS appearing after a laparotomy carried out for small bowel obstruction and after prolonged parenteral feeding.

## 2. Case report

A 64 year old married lady was admitted as an emergency with a four-day history of abdominal pain, constipation and vomiting. She had a past history of appendectomy, hysterectomy, left nephrectomy and laparoscopic cholecystectomy. A clinical diagnosis of small bowel obstruction was supported by X-ray findings and she underwent a laparotomy one-week later. Her alcohol consumption prior to the surgery was thought to be excessive (about 40 units per week), but this could not be confirmed. There was also no evidence of a dependence syndrome.

Post-operatively she was managed on an intensive therapy unit for 24 hours and was transiently hypotensive. She developed a chest infection, and following a central line insertion, a traumatic pneumothorax. Her recovery was slow, and paralytic ileus was present for some weeks and the patient required total parenteral nutrition for approximately one month.

Four weeks after the operation she was noted by the surgical team to be confused and disorientated. An urgent neurological opinion was obtained and it was noticed that in addition to the confusion the patient was confabulating and had marked vertical and horizontal nystagmus. A diagnosis of Wernicke's encephalopathy was made and immediately treatment with intravenous thiamine was undertaken. A red cell transketolase level was low. Cranial computerised tomography on the same day was normal. She received intravenous thiamine (pabrinex) and vitamin (parentrovite) for the initial two weeks period after which she received thiamine 200 mgs twice daily orally with two tablets of multivitamin for the next two months, and thereafter thiamine 200 mgs daily for another 10 months.

Over the subsequent four weeks her confusion became less apparent, and the remaining neurological defect was lateral nystagmus. She still had anterograde amnesia three months post-operation and was transferred to a regional neuropsychiatric centre. Psychological testing soon after her arrival indicated repetitive speech and confabulation, e.g. she told the psychologist that she was currently training to be a doctor.

She performed poorly on the Warrington Recognition Memory Test, both for faces (30/50; at less than 5th percentile-impaired) and words (37/50; at 10th percentile-borderline impairment). Her immediate (5 items) and delayed recall (zero item) on logical memory as tested with the Wechsler Memory Scale-Revised were also poor (at 5th percentile-clear impairment). She also showed evidence of impaired non-verbal memory by performing poorly on drawing from her memory the Rey-Osterrieth complex figure. Her pre-morbid cognitive ability was estimated to be above average as determined by National Adult Reading Test (full scale IQ = 119).

Six weeks later she scored 24 out of 30 in Mini Mental State Examination and just below the threshold level (score of 4) in Luria-Nebraska neuropsychological screening test. She performed well on tests of executive functions such as similarities, explanation of proverbs, alternate sequence drawing and motor sequencing. Other frontal lobe (executive) function test included 'verbal fluency'. On the tests of verbal fluency she performed well within the normal range on category fluency (categories such as animals, fruits, vegetables and supermarket), yet scored at below the normal range on the FAS word fluency test. She still had some residual horizontal (but not vertical) nystagmus at that stage.

Further psychometric assessment occurred three months later. At that stage, her speech was coherent and grammatically correct, although rather repetitive and circumstantial. She was rather vague about current items of news, but was able to mention war in Croatia and Bosnia, and a recent injury sustained by Lynford Christie. Subjectively therefore her memory of recent events appeared better since the onset of Wernicke's encephalophathy, but she showed impairment on the objective cognitive tests. She scored 28 on immediate, 4 on delayed, and 15% on the story recall, and 80 on copy, 36 on immediate, and zero on delayed figure copy test of the Adult Memory and Information Processing Battery. She scored 23 in total (five trials) with two intrusions on Design learning test (Design B). She scored 46 seconds on Trail Making Test A, and 202 seconds in Trail Making Test B. On the Visual Form Discrimination Test she showed 13 correct responses with one peripheral error, one major rotation, and one major distortion.

At this stage, the patient's performance on the visuospatial test was essentially normal. However, she showed deficits on verbal, immediate and delayed recall of non-verbal material, and on non-verbal learning functions. The Trail Making tests also suggested that she had problems with planning and switching attention.

A magnetic resonance imaging (MRI) head scan, using a 1.5-tesla magnet, 10 weeks after surgery showed normal appearance of brain stem and mammillary bodies. Scattered areas of abnormal signal in the cerebral white matter were thought to be age-related ischaemic changes. A single photon emission computed tomogram (SPECT), using a dual-headed Helix gamma camera, two months after the MRI scan showed no bilateral temporal lobe lesion but small foci of reduced perfusion in the right temporo-parietal region. SPECT data were not analysed using any quantitative method.

The patient was discharged from in-patient to day-patient status after a consistent subjective improvement in her cognitive state. She lived with her husband and attended the outpatient department thereafter.

## 3. Discussion

WKS occurs in a state of thiamine deficiency which can be caused by various predisposing and precipitating factors leading to either a decrease in thiamine supply or an increase in the need for thiamine or inefficient absorption through the gastrointestinal tract. Usually WKS arises due to a combination of these factors. This was illustrated in our case report.

The above patient developed the WKS following small bowel obstruction, laparotomy and prolonged parenteral feed. We can hypothesise that the predisposing factors in this case may have been the initial period of anorexia and vomiting leading up to her abdominal operation, the stresses of the operation itself and the subsequent complications, and the limited food intake. The precipitating factor could be the parenteral nutrition without thiamine, possibly causing a further drop in her already low thiamine level.

Various attempts have been made to identify any structural abnormalities associated with the WKS by using structural neuroimaging techniques such as computerised tomography (CT) scan or magnetic resonance imaging (MRI)). Findings, however, are varied and are often non-specific, some of which have not been replicated. In their study, Carlen and colleagues [5] found general cerebral atrophy associated with sulcal widening in WKS patients. These lesions correlated significantly with cognitive impairment. Other findings include significant enlargement of the third ventricle, widening of the Sylvian fissures and the left frontal sulci [36], larger lateral ventricles and wider inter-hemispheric fissures [17], large symmetrical thalamic hypodensity [28], cortical atrophy [7], smaller mammillary bodies [6], bilateral caudate lesions along with symmetrical periventricular lesions of the thalamus and hypothalamus and periaqueductal grey matter [31] and widespread reduction in grey matter size in the anterior part of the diencephalon, the median temporal and orbito-frontal cortex [18]. It is apparent from the structural neuroimaging findings that over and above the specific abnormalities in the temporal lobe and thalamus, there is widespread cerebral atrophy, particularly involving the frontal lobe structures. Although in the current case neuroimaging did not show any evidence of frontal lobe damage, neuropsychological tests showed definite evidence of frontal lobe dysfunction.

Functional neuroimaging studies on WKS patients have shown possible reductions in cerebral blood flow in the hypothalamus and basal forebrain [15], reduced tracer uptake throughout the cortex except for the posterior temporal cortex [16], reduced metabolism in the limbic-hippocampal regions including the thalamic nuclei and frontal basal cortex [11], and anterior brain regions and right posterior white matter with increased activity in cerebellar-cortical connections [25]. It therefore appears that like in the structural neuroimaging studies, no consistent deficit was found in WKS patients using the functional neuroimaging techniques. Studies not only show deficits in specific areas such as temporal lobe and thalamus but a more widespread abnormality including frontal lobe dysfunction has been reported.

In the current case, SPECT scanning revealed reduced perfusion in the right temporo-parietal region, which may explain the patient's impaired non-verbal memory. Surprisingly, MRI scan showed normal appearance of brainstem and mammillary bodies and only scattered ischaemic changes in the white matter, but not the type of lesions we would expect. It could be that other critical lesions were present but too small to be visible on the scan [32]. Atypical lesions with intact thalamus and mammillary bodies and asymmetrical dorsal thalamic dysfunction have been reported [41]. There could also be publication bias in that case reports with positive structural neuro-imaging findings are published more frequently. It also appears that a number of studies show some structural abnormalities which are non-specific [4,35].

This case report serves to illustrate how a serious neuropsychiatric disorder may present following a brief history of small bowel obstruction and poor postoperative feeding. Although there was a suspicion of alcohol abuse, alcohol consumption is not thought to be the primary cause of this patient's disorder. It is, however, possible that in this case the psychopathology may have been a combined effect of alcohol abuse and gastro-intestinal disorder. Long standing alcohol abuse can cause dementia, and widespread cognitive deficits could be associated with WKS. In this case report, there was evidence of verbal and non-verbal memory deficit and frontal lobe dysfunction but no evidence of any neuro-degenerative condition in the form of deteriorating global cerebral function. Small bowel obstruction is a common surgical emergency and the prospect of the WKS following this may indicate that thiamine replenishment should be a prophylactic measure in the peri-operative period.

Previous authors [26,34] have recommended the use of prophylactic thiamine after gastric operations. We do support this recommendation and stress the importance of prophylactic thiamine supplement in the early postoperative period of gastrointestinal surgery. In treatment of malnutrition with parenteral nutrition or during prolonged intravenous administration of fluid, it is equally important to add thiamine prophylactically. There is evidence that the failure to do so can precipitate WKS [29,33], as illustrated by our patient, when intravenous glucose accelerates the consumption of the already depleted thiamine, thereby depleting the thiamine store further. We are of the opinion that one should moreover be vigilant at all times after surgery for any slight indication of WKS as cases of WKS can occur even years after the original index event [37], and treat it promptly with high dose of parenteral thiamine. A delay in doing so, can result in permanent neurological deficits. Shimomura and colleagues [37] have suggested that patients should be educated in proper dietary intake, in carefully monitoring their thiamine intake and in recognising the signs and symptoms of WKS early. It is also important to take extra care in times of increased vulnerability such as prolonged illness and diarrhoea or vomiting, by taking prophylactic thiamine especially if dietary intake is diminished.
